# Facts and Fallacies in the Debate on Glyphosate Toxicity

**DOI:** 10.3389/fpubh.2017.00316

**Published:** 2017-11-24

**Authors:** Robin Mesnage, Michael N. Antoniou

**Affiliations:** ^1^Gene Expression and Therapy Group, Faculty of Life Sciences and Medicine, Department of Medical and Molecular Genetics, King’s College London, Guy’s Hospital, London, United Kingdom

**Keywords:** glyphosate, chronic disease, pesticides, toxicity, glycine, gluten sensitivity, cytochrome P450 enzyme system, microbiome

## Abstract

The safety profile of the herbicide glyphosate and its commercial formulations is controversial. Reviews have been published by individuals who are consultants and employees of companies commercializing glyphosate-based herbicides in support of glyphosate’s reapproval by regulatory agencies. These authors conclude that glyphosate is safe at levels below regulatory permissible limits. In contrast, reviews conducted by academic scientists independent of industry report toxic effects below regulatory limits, as well as shortcomings of the current regulatory evaluation of risks associated with glyphosate exposures. Two authors in particular (Samsel and Seneff) have published a series of commentaries proposing that long-term exposure to glyphosate is responsible for many chronic diseases (including cancers, diabetes, neuropathies, obesity, asthma, infections, osteoporosis, infertility, and birth defects). The aim of this review is to examine the evidential basis for these claimed negative health effects and the mechanisms that are alleged to be at their basis. We found that these authors inappropriately employ a deductive reasoning approach based on syllogism. We found that their conclusions are not supported by the available scientific evidence. Thus, the mechanisms and vast range of conditions proposed to result from glyphosate toxicity presented by Samsel and Seneff in their commentaries are at best unsubstantiated theories, speculations, or simply incorrect. This misrepresentation of glyphosate’s toxicity misleads the public, the scientific community, and regulators. Although evidence exists that glyphosate-based herbicides are toxic below regulatory set safety limits, the arguments of Samsel and Seneff largely serve to distract rather than to give a rational direction to much needed future research investigating the toxicity of these pesticides, especially at levels of ingestion that are typical for human populations.

It doesn’t matter how beautiful your theory is, it doesn’t matter how smart you are. If it doesn’t agree with experiment, it’s wrong. Richard P. Feynman (Nobel Laureate, Physics, 1965)

## Glyphosate: The Controversy Over its Safety

**Figure 1:**
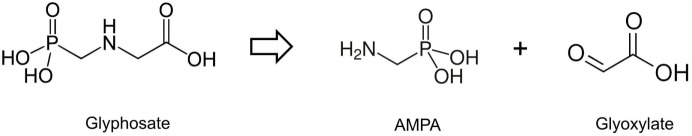
Structure of glyphosate and its breakdown products aminomethylphosphonic acid (AMPA) and glyoxylate.

Glyphosate (*N*-phosphonomethyl glycine) is a small molecule (Figure 1), which acts as an herbicide primarily by inhibiting the enzyme 5-enolpyruvylshikimate-3-phosphate synthase (EPSPS), a key component of the shikimate pathway. Inhibition of the shikimate pathway blocks aromatic amino acid biosynthesis in plants, resulting in their death (1). Glyphosate was patented as an herbicide in 1971 (US Patent No. 3799758). Upon its introduction as an herbicide commercially in 1974, glyphosate quickly became the leading pesticide in the global agrochemical market. Glyphosate sales and use has increased exponentially since 1996 after the introduction of novel glyphosate-tolerant “Roundup-Ready” crop varieties (namely, maize, soybean, canola, cotton, sugar beet, and alfalfa), which are genetically engineered to be sprayed with Roundup without dying (2). The ubiquity of glyphosate in food, water, and air means that it is ingested on a frequent basis and regularly found in human urine at levels around 1–10 µg/L (3).

The effects of glyphosate are well characterized at concentrations and doses causing acute toxicity, with outcomes increasing over time and in a dose-dependent manner. Toxic effects on rat liver, measured after the administration of 60 mg/kg body weight/day of glyphosate over 2 years, formed the basis for calculating the acceptable daily intake (0.3 mg/kg bw/day) within the European Union for the period 2002–2017 (4). However, data regarding health risks arising from ingestion of glyphosate alone at lower than the regulatory permitted daily intake, and which is relevant for human environmental levels of exposure, are far more limited and the subject of much debate (5).

As with any controversial topic, conclusions are not only driven by facts but can also be influenced by commercial or ideological vested interests. Several reviews have been published by individuals who are consultants of companies commercializing glyphosate-based herbicides (6–8) to facilitate the process of glyphosate’s reapproval by regulatory agencies. These authors conclude that glyphosate is safe at levels below regulatory permissible limits. In contrast, reviews conducted by independent scientists based on academia report toxic effects below regulatory limits (5), as well as shortcomings of the current regulatory evaluation of risks associated with glyphosate exposures (9, 10). Other authors have published reviews proposing that long-term exposure to glyphosate is responsible for many chronic diseases (cancers, neuropathies, infections, osteoporosis, etc.) (11–15).

These diverse points of view on glyphosate toxicity has led to extreme discrepancies in the scientific community, has given rise to confusion, and thus deserves clarification. Limitations and recommendations for improvement in the regulatory assessment of the risks to humans from exposure to glyphosate-based herbicides have previously been extensively discussed (9, 10) and will not be detailed here. The aim of this review is to critically evaluate the scientific evidence presented in a series of commentaries used to conclude on the role of glyphosate in the etiology of chronic diseases (11–15).

The five commentaries by Samsel and Seneff propose a link between exposures to environmental levels of glyphosate and the development of a wide range of chronic diseases (11–15). In each commentary, these authors largely construct their arguments on deductive reasoning based on a logistic structure called syllogism, which is formed when two or more propositions are used in order to generate a conclusion. Although syllogisms can help in deductive reasoning, to ensure that they are used in science in a constructive rather than a misleading way, it is necessary to ensure that the two propositions that lead to the conclusion are firmly evidence-based. We therefore evaluated the Samsel and Seneff commentaries to see whether this was indeed the case.

## Can Glyphosate Inhibition of Cytochrome P450 Enzymes and Aromatic Amino Acid Biosynthesis a Cause Chronic Illness?

Their first commentary attempts to make a link between glyphosate ingestion and “most of the diseases and conditions associated with a Western diet” by suppression of the activity of the cytochrome P450 class of enzymes (CYP450) and amino acid biosynthesis by the gut microbiome (11). The claim that glyphosate inhibits the detoxifying CYP450 enzyme system is based on inferences from studies performed on plants or with other pesticides. However, even if some studies do show inhibition of CYP450 at high levels corresponding to agricultural use concentrations (typically 10 g/L of glyphosate), they are not relevant in terms of environmental exposures to which humans are typically exposed (approximately 0.1–1 μg/kg/day) (5). In addition, the authors fail to acknowledge the studies performed in mammals with environmentally relevant levels of Roundup (16), as well as studies on human cell cultures, which actually show an increase in CYP450 activity (17).

Some studies are misrepresented and misquoted in the commentary. For example, the authors refer to a study reporting that the liver of male and female rats exposed to Roundup in their drinking water at glyphosate equivalent levels allowed for human consumption in the US (0.7 mg/L), showed a reduction in CYP450 enzyme levels (18). Samsel and Seneff conclude that this reduction in CYP450 is solely due to glyphosate ignoring the fact that Roundup, which contains a large spectrum of coformulant adjuvants was administered to the animals and not glyphosate alone. It is established that coformulant adjuvants are toxic in their own right resulting in commercial pesticide formulations being more toxic than the stated active ingredient alone (19, 20). Thus, the coformulants may have been responsible either alone or in combination with the glyphosate in the Roundup formulation tested for the observed decrease in rat liver CYP450 levels (18). In addition, the data presented by Larsen and colleagues clearly show that although the global CYP450 content decreased, the activities of the CYP450 enzyme complex in female animals increased (18). Overall, a review of the literature shows that glyphosate and Roundup are likely to increase the activity of CYP450, disproving the conclusions of this first commentary (14).

Samsel and Seneff also postulate that glyphosate disrupts the biosynthesis of aromatic amino acids by gut bacteria, based on a study showing a decrease in amino acid levels in a carrot cell line exposed to glyphosate (21). Although it can be hypothesized that glyphosate may disturb the gut microbiome because some bacteria possess the EPSPS enzyme and shikimate pathway, and thus may indirectly affect aromatic amino acid biosynthesis, this has never been studied in a controlled laboratory animal experiment. Indeed, the patenting of glyphosate as an antibiotic to be used against a wide spectrum of microorganisms was based solely on effects in protozoa (not bacteria) and its effectiveness was dependent on the addition of di-carboxylic acids (US Patent No. 7771736 B2). At this stage, it is currently not clear whether glyphosate has an effect on the mammalian gut microbiome, especially at environmentally relevant levels of exposure. Nonetheless, some studies have shown that glyphosate and glyphosate-based herbicides such as Roundup can selectively affect bacterial populations *in vitro* (22) while others have reported no adverse effects (23). Given these discrepancies additional research is clearly needed to ascertain whether glyphosate-based herbicides at environmentally relevant levels of ingestion can result in disturbances in the gut microbiome of human and animal populations with negative health implications.

## Glyphosate Linked with Rise in Non-Celiac Gluten Sensitivity (NCGS)?

The hypothesis of glyphosate-induced gut microbiome disturbances has led Samsel and Seneff in a second commentary (12) to propose that glyphosate is the most important causal factor in the epidemic of NCGS disease (24). Their arguments are based on the following syllogism. Since glyphosate could have effects on the gut microbiome and since NCGS disease is associated with imbalances in gut bacterial populations, glyphosate could fully explain the etiology of this condition. This syllogism is further extended by these authors by adding that NCGS disease patients have an increased risk of developing non-Hodgkin’s lymphoma and reproductive problems such as infertility, miscarriages, and birth defects, and thus glyphosate could also explain the rise in these latter pathologies (12). Although there have been a number of studies showing an association between occupational glyphosate-based herbicide exposure and non-Hodgkin’s lymphoma and reproductive problems including birth defects (25), a link between typical levels of human exposure and these conditions has not been demonstrated experimentally. It is true that rates of conditions of the gastrointestinal tract such as inflammatory bowel disease have dramatically increased with the adoption of Westernized diets (consumption of processed foods, high in animal protein, processed sugars, starches, and fats) (26). Exposure to increased levels of toxic chemical pollutants could be a crucial factor causing gut microbiome alterations and subsequent gastrointestinal disorders (27). However, a causative link between glyphosate and gut microbiome-associated intestinal disorders remains hypothetical but nonetheless an important area to be investigated.

## Is Glyphosate Chelation of Manganese a Cause of Chronic Illnesses?

In their third commentary, Samsel and Seneff create multiple syllogisms based on the fact that glyphosate can chelate manganese (Mn) (13). At face value, there is merit in this supposition, since glyphosate was originally patented and used as a divalent cation metal chelator (US Patent No. 3160632A). These authors propose that the dysregulation of Mn homeostasis by glyphosate chelation could cause osteoporosis and osteomalacia (because bone mineralization depends on Mn), seizures (associated with reduced serum Mn), and prion diseases (since the prion protein, PrP, can misfold following binding to Mn instead of Cu). They also claim that large-scale environmental damage, such as the collapse of coral reefs, may in fact be due to glyphosate because coral mucus contains sulfated glycoproteins similar to chondroitin sulfate, whose synthesis is dependent on Mn. However, the conclusions from this commentary are speculative since the effects of glyphosate on metal micronutrient homeostasis have never been characterized. Samsel and Seneff propose that glyphosate chelation of Mn can promote binding of this nutrient metal to PrP, causing it to misfold, and rendering it capable of catalyzing metal-free aggregation of this protein (28), which in turn could lead to prion disease. However, the sequestration of Mn by glyphosate would effectively make it unavailable to participate in interactions with other substances including proteins, making it unable to bind in place of Cu to PrP to promote misfolding and prion disease as suggested. Indeed, based on the arguments presented, chelation of Mn by glyphosate would be protective against, rather than a causative agent of, prion disease as this would prevent this divalent cation from binding to PrP.

Out of the 328 references quoted in these authors’ third commentary (13), which are used to support their proposal of a link between Mn chelation by glyphosate and chronic diseases, only one study reports the effects of glyphosate on Mn levels in animals (29). This investigation looked at a possible connection between urinary concentrations of glyphosate and Mn, and health indicators in Danish dairy cows. The results revealed a correlation between markers indicative of a disturbance in kidney function and glyphosate urinary concentration; i.e., the higher the levels of glyphosate found in the urine, the greater the indicators of kidney dysfunction. However, although Mn levels were abnormally low, they were not correlated with urinary glyphosate levels. Although no doubt interesting additional studies are required to clarify the mechanism of the observed low levels of Mn in these farm animals. Thus, this study (29) cannot be used to conclude on an effect of glyphosate on Mn homeostasis. The conclusions of the third commentary by Samsel and Seneff are thus unsupported by evidence.

## Is Glyphosate Responsible for the Steep Rise in Certain Cancers?

The fourth commentary by Samsel and Seneff discusses the question of the carcinogenic potential of glyphosate (14). Conclusions are based on correlations between time trends in various cancers and glyphosate-based herbicide application on corn and soy crops. The reason given is that it appears that increasing cancer rates reported by the US Centers for Disease Control and Prevention from 1990 and 2010 parallel the dramatic increase in the volume of glyphosate-based herbicide application on these crops due to introduction of varieties in 1996 that are genetically engineered to tolerate being sprayed with this pesticide (2). Although it is surprising to see that the trends in increased glyphosate-based herbicide use and increasing incidence of certain cancers closely overlap, conclusions on a causative link between the two ignores two fundamental facts. First, the vast increase in the use of glyphosate-based herbicides due to expansion in the cultivation of glyphosate-tolerant genetically engineered crops did not become a substantial proportion of US agriculture until the turn of the century, with 66% of the total volume of glyphosate applied in the US from 1974 to 2014 taking place between 2004 and 2014 (2). Thus, significant increases in exposure of human populations to glyphosate have also only occurred since the year 2001. Second, it is well established that there is always a delay or lag period between exposure to a carcinogen and formation and detection of a cancer, with this delay varying depending on the type of cancer. Thus, the fact that increases in glyphosate-based herbicide use overlap with, for example, an increase in breast cancer incidence is more indicative of an absence of a connection rather than a link between the two phenomena. Thus, the increase in the use of glyphosate, and thus exposure to this compound, and the etiology of cancer are both out of step with the proposed link of this herbicide with cancer causation. In addition, cancer can be caused by a myriad of factors, some known and most unknown. Thus, statistical correlations of cancer incidence with exposure to a specific agent is insufficient to establish a causal link.

The known biology of cancer suggests that we look further back in history to identify the causative factors that have led to the steep increase in this class of diseases starting in the mid-1990s.

This fourth commentary (14) also discusses the World Health Organization’s International Agency for Research on Cancer (IARC) classification of glyphosate as a probable (Group 2A) human carcinogen (30). However, this IARC categorization of glyphosate has no bearing on, and thus cannot be used to support, the principal message of this commentary, which is that the increased incidence of some cancers has paralleled the escalation in use of glyphosate-based herbicides since the mid-1990s and thus suggests a causative link. The IARC assessment and scoring of glyphosate as a Group 2A carcinogen is based on sufficient evidence of carcinogenicity in animals (30), limited evidence of carcinogenicity in humans (increased rates of non-Hodgkin lymphoma in farmers) (30), and strong mechanistic evidence (genotoxicity and oxidative stress) (30).

## Can Glyphosate Substitute for Glycine in Polypeptide Chains?

In their fifth and latest commentary, Samsel and Seneff present what is arguably their most radical hypothesis regarding mechanisms of glyphosate toxicity (15). The core message of this publication is that glyphosate, being a derivative of glycine, can substitute for the native amino acid in proteins. Based on this supposition, it is postulated that such mis-incorporation of glyphosate in place of glycine can lead to polypeptide chain misfolding and aberrant cellular biochemistry that could lead to disease. By this mechanism, the authors argue a link between glyphosate exposure and an extremely large spectrum of disease conditions, including diabetes, obesity, asthma, chronic obstructive pulmonary disease, pulmonary edema, adrenal insufficiency, hypothyroidism, Alzheimer’s disease, amyotrophic lateral sclerosis, Parkinson’s disease, prion diseases, lupus, mitochondrial disease, non-Hodgkin’s lymphoma, neural tube defects, infertility, hypertension, glaucoma, osteoporosis, fatty liver disease, and kidney failure. However, a number of the conceptual and experimental tenets used by Samsel and Seneff to assert that glyphosate can substitute for glycine in proteins are flawed.

First, Samsel and Seneff argue that since glyphosate can potentially form *N*-substituted glycine polymers known as peptoids (31), then it can also replace glycine in regular polypeptides. However, as peptoids are laboratory creations that do not exist naturally in living organisms, it is not valid to extrapolate from these laboratory-manufactured entities to suggest that glyphosate can substitute for glycine in naturally occurring polypeptides, which are biosynthetically and structurally distinct from peptoids. In this context, it is perhaps also noteworthy that to the best of our knowledge, there are no reports of glyphosate peptoids having been generated.

Second, Samsel and Seneff quote in support of their arguments results from studies conducted by scientists at DuPont, a company based in the US. The references provided [numbers 34 and 35 in Samsel and Seneff (17)] are company reports dating back to 2007, which have not been published in the peer-reviewed scientific literature and thus are unavailable for scrutiny to verify the conclusions drawn from these investigations. Nevertheless, Samsel and Seneff make the following arguments, which they claim provide strong evidence of glyphosate’s incorporation into proteins in place of glycine. First, they state that only 42% of the radioactively labeled ^14^C-glyphosate administered to goats was extractable from muscle from these animals and that treatment with pepsin and an additional (undisclosed) protease did not release any additional ^14^C-glyphosate from this tissue. In their view, this suggests that the ^14^C-glyphosate had been incorporated into proteins and thus was non-extractable by the methods used. In addition, they state that to more fully release ^14^C-glyphosate from the liver, kidney, and omental fat of goats or the eggs of chickens fed with this substance, required treatment with pepsin, which again in their view suggests that the glyphosate had been “incorporated” into proteins in place of glycine. These arguments not only ignore the apparent contradiction in their statements (pepsin/protease treatment of tissues does/does not lead to ^14^C-glyphosate release) but also a far simpler and more likely reason for these observations; i.e., the glyphosate was either adsorbed onto or trapped within protein structures/aggregates and is liberated upon protease digestion.

In contrast to the proposed notion that glyphosate can substitute for glycine in polypeptide chains, it has recently been reported that a very high dose (200 mg/kg) of this compound administered to mice is metabolized by being cleaved into aminomethylphosphonic acid and glyoxylate (Figure 1), with the latter covalently modifying certain amino acids, for example, cysteine, within proteins (32). It is important to note that it is not glyphosate that is able to post-translationally modify proteins but its breakdown product glyoxylate. The very high, non-environmentally relevant dose administered is acknowledged by the authors of this study, who rightly conclude that the implications of their findings to human health are unknown.

It also needs to be borne in mind that the incorporation of glyphosate into proteins during translation would be in direct competition with glycine. Since the translation machinery has evolved to function with glycine and not glyphosate, then the incorporation of the latter into proteins would be, in all likelihood, a very inefficient process. Furthermore, if glyphosate were to be incorporated into proteins, causing them to become mis-folded, then these in most instances would be recognized as abnormal, resulting in their ubiquitination and degradation.

Finally, direct experimentation has shown that glyphosate does not incorporate into proteins (33). In these studies, *E. coli* were cultured in the presence of high concentrations (1 g/L) of glyphosate and rescued by addition of aromatic amino acids in the culture medium. Analysis of these bacteria showed that they possessed proteins with the exact same molecular weight as in non-exposed control cultures (33), demonstrating that glyphosate could not have been incorporated in place of glycine. Alternatively, the culture of *E. coli* in the presence of glyphosate and 6-fluorotryptophan resulted in polypeptides containing this variant aromatic amino acid and no incorporation of glyphosate (34). If glyphosate had been incorporated into the proteins of these bacteria, a shift in protein molecular weight would have occurred and would have been readily detectable by the sensitive and accurate mass spectroscopic analytical methods employed in these studies (33, 34).

In summary, the arguments used by Samsel and Seneff do not provide evidence for the substitution of glycine by glyphosate within proteins; on the contrary, there is good experimental evidence that implies that this does not take place.

## Discussion

Overall, a scrutiny of the method used in these commentaries by Samsel and Seneff reveals a major flaw. These authors employ a deductive reasoning approach based on syllogism, which is formed by two or more propositions used to generate a conclusion. The first proposition is generally related to glyphosate’s properties (e.g., glyphosate is a chelator of Mn) and the second proposition is related to human physiology (e.g., sperm motility depends on Mn). From each of these pairs of propositions, Samsel and Seneff conclude a causative link of glyphosate with the etiology of different diseases. For instance, since glyphosate is a metal chelator (proposition 1), and since sperm motility depends on Mn (proposition 2), they conclude that glyphosate may partially explain increased rates of infertility and birth defects (13). They extend this reasoning to multiple body functions to propose that the dysregulation of Mn utilization in the body due to glyphosate’s metal chelating properties explains autism, Alzheimer’s disease, Parkinson’s disease, anxiety disorder, osteoporosis, inflammatory bowel disease, renal lithiasis, osteomalacia, cholestasis, thyroid dysfunction, and infertility. More recently, Beecham and Seneff have used the same reasoning to conclude on a causative link between glyphosate chelation of Mn and the large rise in the incidence of autism spectrum disorders in children within the US (35). However, there are no scientific studies establishing a causative link between glyphosate and the described chronic diseases.

Furthermore, again using the same syllogism structure methodology Seneff and Nigh have suggested that the chelation by glyphosate of nutrient metals, specifically Zn and Co, could lead to developmental and metabolic disturbances that are responsible for increasing the risk of anencephaly (36). However, as in the case of Mn, there is no evidence to suggest that glyphosate at typical levels of daily ingestion disrupt Zn and Co homeostasis that could contribute to this increasing the risk of giving birth to an anencephalytic child.

Syllogisms can help in deductive reasoning, but they can also be used to form incorrect conclusions, which are known as syllogism fallacies. Thus, to ensure that syllogisms are used in a constructive rather than a misleading manner, it is necessary to ensure that the two propositions that lead to the conclusion are firmly evidence-based and make biological sense. Frequently quoted trivial but informative examples used to illustrate these points are “All men are mortal, Socrates is a man, therefore Socrates is mortal,” constitutes a meaningful syllogism whereas “All cats are mortal, Socrates is mortal, therefore Socrates is a cat” is clearly a syllogism fallacy! The syllogisms employed by Samsel and Seneff do not appear to have a solid evidential basis and thus could be considered misleading.

The underestimation of the toxicity of a commercialized product is known to have devastating effects on public health. Although it has long been asserted by both industry and regulatory agencies that glyphosate is safe even at relatively high daily intake levels (for example, 1.75 mg/kg bw/day in the US), major gaps in its evaluation have been identified and need to be addressed in order to definitely conclude on its safety (9, 10). For example, glyphosate has never been tested alone at its acceptable daily intake or at doses relevant for human exposures. Only recently have studies been published that reveal kidney and especially liver structure and functional damage in rats following chronic ingestion of an ultra-low, environmentally relevant dose of a glyphosate-based herbicide (Roundup) (37, 38). In addition, major endpoints of toxicity, such as developmental, reproductive, transgenerational, and even chronic effects in adults still need to be investigated under controlled laboratory animal conditions, at environmentally relevant doses, using feed and water free from incidental glyphosate contamination. Indeed, most glyphosate toxicity studies have been performed without controlling for glyphosate contamination in food or water of laboratory animals used as “non-exposed” controls, even though this feed has been found to be regularly contaminated by glyphosate residues (39).

## Conclusion

Our critical analysis of the commentaries published by Samsel and Seneff reveals that their conclusions are not substantiated by experimental evidence but are based on a type of failed logic known as syllogism fallacies. As Nobel Prize-winning theoretical physicist Richard Feynman famously said, “*It doesn’t matter how beautiful your theory is, it doesn’t matter how smart you are. If it doesn’t agree with experiment, it’s wrong*.” In this regard, the mechanisms and vast range of conditions proposed to result from glyphosate toxicity presented by Samsel and Seneff in their commentaries are at best unsubstantiated theories or speculations that are not supported by experimental observation and thus are likely to be wrong. This misrepresentation of glyphosate’s toxicity could waste a large amount of time on the part of regulators, industry, and the concerned public, tying up resources that should be used to follow up more solidly based lines of investigation as previously suggested (9, 10).

## Author Contributions

RM and MA wrote the review together.

## Conflict of Interest Statement

The authors declare that the research was conducted in the absence of any commercial or financial relationships that could be construed as a potential conflict of interest.
